# Supplementary material for: Amino Acid Metabolic Signature Links Pediatric Environmental Enteric Dysfunction to Maternal-Fetal Growth Restriction Across Two Independent Cohorts
Source: bioRxiv. 2026 Oct 30:2026.10.25.760166. Preprint. [Version 1] doi: 10.64898/2026.10.25.760166 (PMC13733850; doi:10.64898/2026.10.25.760166)
Supplement: Supplementary file 1 [file NIHPP2026.10.25.760166V1-supplement-1.pdf]

bioRxiv preprint doi: https://doi.org/10.64898/2026.10.25.760166; this version posted October 30, 2026. The copyright holder for this preprint (which was not certified by peer review) is the author/funder, who has granted bioRxiv a license to display the preprint in perpetuity. It is made available under a CC-BY 4.0 International license.

Supplementary Methods

Sample Collection

Cohort A (AMANHI)

Biological material collected from Cohort A participants included maternal blood, urine, and stool samples, placental tissue, and infant cord blood. Trained phlebotomists collected maternal and umbilical cord blood, which was centrifuged to obtain serum, plasma, and buffy-coat aliquots. Maternal antenatal blood and urine samples were collected at a single timepoint per participant. with women randomized to sample collection at either the 24–28 week or 32–36 week antenatal visit. Placental and umbilical cord tissue were collected at delivery, with a portion flash-frozen and a portion fixed for histological examination. Maternal and newborn stool samples were collected for microbiota analysis. All biospecimens were processed and stored at −80°C following standardized, harmonized protocols implemented across AMANHI sites, and biobanked at the Aga Khan University, Karachi, Pakistan for the Karachi cohort.

Cohort B (SEEM)

Duodenal tissue was utilized for bulk RNA sequencing. Due to ethical and logistical constraints precluding obtaining duodenal biopsies from healthy children in this setting, age-matched comparator biopsies were obtained from children undergoing endoscopy at CCHMC for unrelated clinical indications, restricted to those whose biopsy findings and final diagnosis did not support eosinophilic esophagitis, celiac disease, or inflammatory bowel disease, and who had not received antibiotics within four weeks of endoscopy.

Definition of Growth and Nutritional Status Outcome Groups (EED Proxy Phenotypes)

Birth-Defined Outcomes

bioRxiv preprint doi: https://doi.org/10.64898/2026.10.25.760166; this version posted October 30, 2026. The copyright holder for this preprint (which was not certified by peer review) is the author/funder, who has granted bioRxiv a license to display the preprint in perpetuity. It is made available under a CC-BY 4.0 International license.

Three proxy phenotypes were defined using data collected at delivery. 1) Preterm birth, defined as delivery prior to 37 completed weeks of gestation, was compared against term delivery (≥ 37 completed weeks of gestation); 2) Small-for-gestational-age (SGA), defined as a birth weight-for-gestational-age percentile below the 10th percentile, was compared against appropriate-for-gestational-age (AGA) infants (birth weight-for-gestational-age percentile ≥ 10th percentile). 3) Low birth weight (LBW), defined as a birth weight below 2.5 kg regardless of gestational age, was compared against normal birth weight (NBW) infants (birth weight ≥ 2.5 kg).

Follow-up-Defined Outcomes

Two proxy phenotypes were defined using anthropometric data collected at the 24-month follow-up visit: 1) Stunting, defined as a height-for-age Z-score (HAZ) below −2.0 relative to the WHO Child Growth Standards, was compared against non-stunted children (HAZ ≥ −2.0). 2) Wasting, defined as a weight-for-height Z-score (WHZ) below −2.0 relative to the WHO Child Growth Standards, was compared against non-wasted children (WHZ ≥ −2.0).

Biospecimens

Cohort A (AMANHI) - Plasma Proteomics

A total of 100 µL of maternal plasma per sample (two aliquots per participant) was shipped from Aga Khan University, Pakistan, to the Max Planck Institute of Biochemistry, Germany, for untargeted proteomic analysis. From each sample, 5 µL of plasma was processed using a perchloric acid workflow with neutralization (PCA-N), which significantly reduces costs with greater proteomic depth. (13). Details of the full proteomics workflow are included in supplemental methods.

bioRxiv preprint doi: https://doi.org/10.64898/2026.10.25.760166; this version posted October 30, 2026. The copyright holder for this preprint (which was not certified by peer review) is the author/funder, who has granted bioRxiv a license to display the preprint in perpetuity. It is made available under a CC-BY 4.0 International license.

The PCA-N workflow was semi-automated using a Bravo liquid handling platform. Plasma (5 µL) was diluted in 25 µL of water, combined with perchloric acid, and incubated in 96-well plates. Following centrifugation, the supernatant was transferred to a 384-well plate and neutralized to a pH of 8.0–8.5 to provide optimal conditions for enzymatic digestion. Subsequently, proteins were reduced, alkylated, denatured and digested using trypsin.

Following digestion, peptides were loaded onto disposable Evotip C18 trap columns using the Bravo liquid handling platform and separated using an Evosep One liquid chromatography system operated in the 60 samples-per-day (SPD) method. This was coupled to an Orbitrap Astral mass spectrometer (Thermo Fisher Scientific) which employed data-independent acquisition (DIA) technique. Raw DIA data were processed with DIA-NN 1.8.1 against a spectral library predicted in silico with AlphaPeptDeep from the human reference proteome (UniProt UP000005640), and protein-level intensities were obtained with directLFQ and log2-transformed.

Cohort B (SEEM) - Duodenal Tissue Transcriptomics

RNA was extracted from duodenal biopsy tissue collected at endoscopy from 52 children with Environmental Enteric Dysfunction (EED) and 24 comparator controls. Sequencing libraries were prepared using polyA RNA selection, followed by fragmentation, complementary DNA (cDNA) synthesis, and adaptor ligation, using the TruSeq RNA Sample Preparation kit (Illumina). Libraries were sequenced as paired-end 75 base pair reads on an Illumina HiSeq 2000 platform at the Cincinnati Children's Hospital Medical Center (CCHMC) Digestive Health Center Gene Expression Core, with a minimum sequencing depth of 20 million reads per sample (6, 11).

Raw sequencing reads (FASTQ files) were processed using a standardized, cloud-based RNA-sequencing pipeline (37). Reads were aligned to the human reference genome using the

bioRxiv preprint doi: https://doi.org/10.64898/2026.10.25.760166; this version posted October 30, 2026. The copyright holder for this preprint (which was not certified by peer review) is the author/funder, who has granted bioRxiv a license to display the preprint in perpetuity. It is made available under a CC-BY 4.0 International license.

STAR aligner, and gene-level transcript counts were subsequently quantified for use in downstream context-specific metabolic modeling.

Statistical and Bioinformatic Analysis

Generic Genome-scale Metabolic Model (Human1)

Genome-scale Metabolic Models (GEMs) are mathematical models of the human cell which use Gene-Protein-Reaction association rules to link genomic, transcriptomic, or proteomic data to their catalyzed metabolic reactions. GEMs are intentionally generic, able to complete a core list of metabolic functions that any cell should. Several popular GEMs are widespread in literature such as Recon3D (28) and Human1 (29). Our analysis uses Human1 for its rich, open-source community and extensive documentation, although the methods described could theoretically be applied to any similar GEM.

Reactome

Reactome (30) is an open-source database of curated, peer-reviewed metabolic pathways. Reactome provides built-in analysis tools for Pathway Enrichment Analysis, determining statistically over-represented pathways from an input list of genes or proteins. We applied Reactome's analysis tools to over- and under-abundant proteins to identify affected metabolic pathways.

Enrichr

Enrichr (31) is a large collection of gene set libraries and is a superset of Reactome. Beyond pathways, Enrichr maps genes to biological processes, diseases, transcription factors, and more. We employed the use of Enrichr libraries to elucidate relevant biological processes related to differentially abundant proteins.

Reaction Inclusion by Parsimony and Transcript Distribution (RIPTiDe)

bioRxiv preprint doi: https://doi.org/10.64898/2026.10.25.760166; this version posted October 30, 2026. The copyright holder for this preprint (which was not certified by peer review) is the author/funder, who has granted bioRxiv a license to display the preprint in perpetuity. It is made available under a CC-BY 4.0 International license.

RIPTiDe (32) is an algorithm based on parsimonious Flux Balance Analysis (pFBA) that, given transcriptomic evidence, prunes a large, generic metabolic model to produce a smaller, context-specific metabolic model. RIPTiDe was used for creating context-specific metabolic models based on SEEM transcriptomic or AMANHI proteomic data. Unlike methods that seek maximum consensus with ‘omics data, RIPTiDe first applies an ‘omics-agnostic pFBA approach to maximize the flux through an objective reaction (typically a generic biomass or cell maintenance reaction) and then measures the correlation of the resulting contextualized model to the ‘omics data. This method has been demonstrated to more accurately predict real metabolic activity in *Escherichia Coli* and has been used in similar studies (5, 33), justifying our usage here. Other contextualization algorithms like Cost Optimization Reaction Dependent Assessment (CORDA) (34) or Fast Task-driven Integrative Network Inference for Tissues (ftINIT) (35) are used for proteomics data integration, but require the user to set a known expression threshold a priori or bin proteins in high, medium, and low expression levels (losing much of the granularity that our quantitative proteomics data provides).

Linear Discriminant Analysis (LDA)

LDA is a supervised machine learning technique that identifies the linear decision boundary that maximally separates the classes of interest. In our metabolic modeling analysis, this technique was chosen to identify the most important reaction-level differences in context-specific metabolic models.

Data Pre-Processing

Plasma proteomic samples missing values for more than 40% of proteins, and proteins missing values in more than 10% of samples, were excluded from analysis. Remaining missing values were imputed using Iterative Singular Value Decomposition (SVD) (36) with a rank empirically determined to minimize a bootstrapped estimate of the matrix reconstruction error. To confirm

bioRxiv preprint doi: https://doi.org/10.64898/2026.10.25.760166; this version posted October 30, 2026. The copyright holder for this preprint (which was not certified by peer review) is the author/funder, who has granted bioRxiv a license to display the preprint in perpetuity. It is made available under a CC-BY 4.0 International license.

that imputation did not introduce statistical bias, the distributions of each protein's abundance values were compared before and after imputation using a two-sample Kolmogorov-Smirnov test, with a Bonferroni correction applied at a significance threshold of 0.05. Following imputation and post-hoc validation, plasma samples from the third antenatal visit (32–36 weeks gestation) were excluded from downstream analysis due to low sample size. To strengthen downstream comparisons, analysis was further restricted to participants with plasma samples available at both the first (8–19 weeks) and second (24–28 weeks) antenatal visits.

Differential Expression Analysis

Protein-level differences between maternal plasma proteomic subcohorts were identified using Mann-Whitney U tests with a Benjamini-Hochberg correction, at a significance threshold of 0.05. A protein was considered differentially abundant between two subcohorts if its abundance distributions differed significantly and the absolute log2 fold change was ≥ 1.0.

Pathway Enrichment and Over-Representation Analysis

To infer biological processes underlying differential protein abundance, over-representation analysis (ORA) was performed using Reactome and Enrichr. Sets of significantly upregulated and downregulated proteins were analyzed against Reactome pathways and Enrichr libraries to identify enriched molecular processes. If no such proteins existed, the log2 fold change was relaxed from 1.0 to ≈ 0.14 (equivalent to a 10% fold change) to permit analysis with Reactome and Enrichr. Enrichment significance was assessed using multiple testing-adjusted p-values (FDR). This enabled orthogonal interrogation of dysregulated pathways, strengthening confidence in pathway-level inference.

Random Forest Modeling

bioRxiv preprint doi: https://doi.org/10.64898/2026.10.25.760166; this version posted October 30, 2026. The copyright holder for this preprint (which was not certified by peer review) is the author/funder, who has granted bioRxiv a license to display the preprint in perpetuity. It is made available under a CC-BY 4.0 International license.

Random forests are a popular machine learning method that extend decision trees to reduce variance and provide robust predictions. To measure the value of maternal metadata in predicting growth proxies, we trained random forest models on maternal metadata (without proteomics) and measured the Out-Of-Bag (OOB) balanced accuracies as a performance metric.

Sensitivity Analysis

A subcohort of the AMANHI participants who had at least one comorbidity (chronic hypertension, diabetes, epilepsy, tuberculosis, thyroid disease) were investigated for their potential impact on the larger cohort. Fisher's Exact Tests with 10 million rollouts each and a Benjamini-Hochberg correction were applied to identify significant (FDR < 0.05) associations between these comorbidities and our five growth proxies.

We also conducted a sensitivity analysis by excluding these participants from the larger AMANHI cohort and repeating the metabolic modeling methods described above. The reactions identified from the sensitivity analysis were compared with reactions identified using the entire cohort to study the influence of these participants on the overall pipeline and characterize the pipeline's sensitivity to anomalies.

Ethical Approval and Consent

SEEM was approved by the Aga Khan University Ethical Review Committee (ERC: 2446 Ped ERC-13) and the Institutional Review Board at Cincinnati Children's Hospital Medical Center (CCHMC, Study ID 2016-0387), and is registered with ClinicalTrials.gov (NCT03588013). AMANHI was approved by Aga Khan University Ethical Review Committee as well as by the World Health Organization Ethics Review Committee (RPC 532). Written informed consent was

bioRxiv preprint doi: https://doi.org/10.64898/2026.10.25.760166; this version posted October 30, 2026. The copyright holder for this preprint (which was not certified by peer review) is the author/funder, who has granted bioRxiv a license to display the preprint in perpetuity. It is made available under a CC-BY 4.0 International license.

obtained from all participants (or their parent/legal guardian, for pediatric participants) prior to enrollment in each study.

Data and Code Availability

All figures were generated and analysis was performed in Python 3.14.3 using libraries including conda (26.7.2), pip (26.0.1), numpy (2.4.6), pandas (2.3.3), cobra (0.31.1), fisher-rxc (1.0.2), matplotlib (3.10.9), riptide (3.4.81), seaborn (0.13.2), statannotations (0.7.2), gurobi (13.0.2), scikit-learn (1.8.0), umap-learn (0.5.12), scipy (1.17.1), fancyimpute (0.7.0), and tqdm (4.67.3). Additional information required to re-analyze the reported data is available from the lead author on request.

The code written to conduct the analysis is available at https://github.com/SyedLab-GI/CompEE

Transcriptomic data for the SEEM study is available at GEO Accession code GSE159495.

Proteomic data for the AMANHI study is not publicly available, but may be requested from the lead author (FJ, and IN).

Supplementary Results

Proteomics Data Pre-Processing

Iterative SVD with a rank of 66 was found to minimize a bootstrapped estimate of the matrix reconstruction error for imputing missing proteomics values. The distribution of five proteins were found to be significantly (FWER < 0.05) affected by a two-sample Kolmogorov-Smirnov test. However, upon investigation, the effect sizes were all negligible (between 0.05 and 0.06, allaying concerns of bias from imputation.

bioRxiv preprint doi: https://doi.org/10.64898/2026.10.25.760166; this version posted October 30, 2026. The copyright holder for this preprint (which was not certified by peer review) is the author/funder, who has granted bioRxiv a license to display the preprint in perpetuity. It is made available under a CC-BY 4.0 International license.

2,458 plasma samples were collected at visit 1, 1,890 at visit 2, and 235 were collected at visit 3. Only the 1,864 participants who had plasma samples collected at both visits 1 and 2 were retained for differential expression, pathway enrichment, and metabolic modeling analyses.

Differentially Abundant Proteins Across EED Proxy Comparisons

Differential Expression Analysis revealed statistically significant differences in protein abundance between SGA and AGA, LBW and NBW, Stunted at 2 Years and Not Stunted at 2 Years, and Wasted at 2 Years and Not Wasted at 2 Years cohorts. However, none of these differences met the log2 fold change threshold of 1.0 for biological significance. No significant differences (either statistically or biologically) were found for the Preterm and Term cohort.

Pathway Enrichment Analysis

Since no proteins met the log2 fold change threshold, the threshold was relaxed to ≈ 0.14 (equivalent to a 10% fold change) for downstream pathway enrichment analysis. Exploratory ORA conducted via Reactome and Enrichr identified up-regulation of immune activation and tissue remodeling and down-regulation of barrier integrity and cellular homeostasis in mothers whose children were SGA, up-regulation of Extracellular Matrix (ECM) remodeling in the LBW cohort, up-regulated inflammatory response and decreased lymphocyte migration in the stunting cohort, and increased cell adhesion in the wasting cohort.

Predicting Growth Proxies from Maternal Metadata

Across all five proxies, the Random Forest models exhibited near coin-flip accuracy, indicating that, without proteomics data integration, maternal metadata alone was an insufficient predictor of these growth proxies **(Supplementary Table 2).**

Sensitivity Analysis

bioRxiv preprint doi: https://doi.org/10.64898/2026.10.25.760166; this version posted October 30, 2026. The copyright holder for this preprint (which was not certified by peer review) is the author/funder, who has granted bioRxiv a license to display the preprint in perpetuity. It is made available under a CC-BY 4.0 International license.

7.8% of mothers in the AMANHI cohort (n = 194) had a history of comorbidities known to adversely affect pregnancy (chronic hypertension (n = 140), diabetes (n = 19), epilepsy (n = 2), tuberculosis (n = 32), or thyroid disease (n = 12)). Categorical associations between presence of each comorbidity and each of the five proxies as well as pregnancy outcome (live birth, stillbirth, or miscarriage) were tested. No significant (FDR < 0.05) associations were found **(Supplementary Table 1)**.

This subcohort was further interrogated using the differential expression, pathway enrichment, and metabolic modeling pipelines utilized for the five proxies of EED described earlier. Differential expression again revealed statistically – but not biologically – significant differences in protein abundance in mothers with one or more of these comorbidities. After relaxing the log2 fold change threshold to ≈ 0.14, analysis of upregulated and downregulated proteins revealed affected immune system activity, hemostasis, metabolism of carbohydrates and carbohydrate derivatives, and metabolism of water-soluble vitamins. Metabolic modeling with RIPTiDe and Human1 primarily implicated Vitamin A metabolism and Amino Acid Metabolism in the top 10 identified reactions, corroborating the pathway enrichment analysis findings.

To identify the impact of these participants on the larger cohort, a sensitivity analysis was conducted wherein the differential expression, pathway enrichment, and metabolic modeling analyses were repeated with these 194 participants removed. Although the specific reactions identified by LDA, the subsystems associated with the top 10 reactions remained highly similar, validating the pipeline’s resilience against outliers.

bioRxiv preprint doi: https://doi.org/10.64898/2026.10.25.760166; this version posted October 30, 2026. The copyright holder for this preprint (which was not certified by peer review) is the author/funder, who has granted bioRxiv a license to display the preprint in perpetuity. It is made available under a CC-BY 4.0 International license.

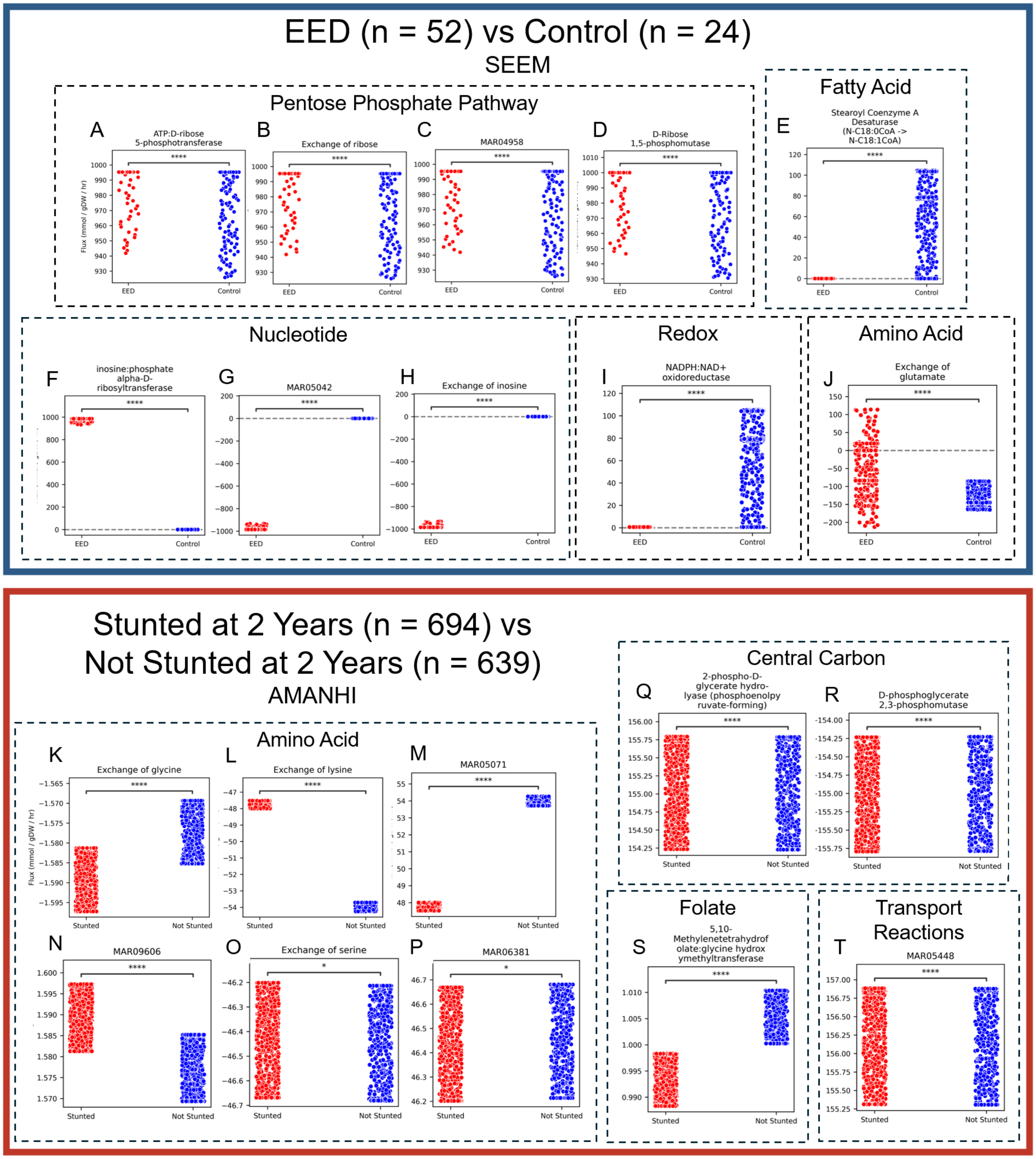

bioRxiv preprint doi: https://doi.org/10.64898/2026.10.25.760166; this version posted October 30, 2026. The copyright holder for this preprint (which was not certified by peer review) is the author/funder, who has granted bioRxiv a license to display the preprint in perpetuity. It is made available under a CC-BY 4.0 International license.

Supplementary Figure 1. Altered Amino Acid Metabolism Differentiates EED from Controls and Stunted from Not Stunted.
Jitter plots showing the top 10 reactions identified by our RIPTiDe and LDA framework. Our framework was applied to SEEM duodenal transcriptomics (A-J) and to AMANHI maternal plasma proteomics from visit 2 (24-28 weeks) (K-T). The top 10 reactions were categorized into subsystems based on each reaction's biochemical function by literature review.. In each plot, the x-axis describes the altered reaction between 1) EED (n = 52) or 2) Stunted (n = 694) and 1) controls (n = 24) or 2) Not Stunted (n = 639). The y-axis shows the flux values sampled from the context-specific metabolic network reconstructions produced by RIPTiDe. A two-sided Mann-Whitney U-test was used to assess the significance of differences in flux distributions. Note that the scale of the y-axes varies between plots and is shown primarily for illustrative purposes.

Both sets of discriminating reactions implicate Amino Acid Metabolism (J, K, L, M, N, O, P).

n.s. = not significant (p >= 0.05)
* = 0.01 < p < 0.05
** = 0.001 < p < 0.01
*** = 0.0001 < p < 0.001
**** = 0.00001 < p < 0.0001

Abbreviations: EED = Environmental Enteric Dysfunction; SEEM = Study of Environmental Enteropathy and Malnutrition; AMANHI = Alliance for Maternal and Newborn Health Improvement; MAR04958 = ribose [c] ⇔ ribose [e]; MAR05042 = inosine [c] ⇔ inosine [e]; MAR05071 = lysine [e] ⇒ lysine [c]; MAR09606 = H^+ [c] + Na^+ [e] + glycine [e] ⇒ H^+ [e] + Na^+ [c] + glycine [c]; MAR06381 = H^+ [c] + Na^+ [e] + serine [e] ⇒ H^+ [e] + Na^+ [c] + serine [c]; MAR05448 = HCO_3^- [c] + 2 Na^+ [c] ⇒ HCO_3^- [e] + 2 Na^+ [e]; RIPTiDe = Reaction Inclusion by Parsimony and Transcript Distribution; LDA = linear discriminant analysis; ATP = adenosine triphosphate; NADPH = nicotinamide adenine dinucleotide phosphate; NAD+ = nicotinamide adenine dinucleotide; H^+ = hydrogen ion; Na^+ = sodium ion

Definitions: Stunted = Length-for-Age Z-Score (LAZ) < -2.0; Not Stunted = Length-for-Age Z-Score (LAZ) ≥ -2.0.

bioRxiv preprint doi: https://doi.org/10.64898/2026.10.25.760166; this version posted October 30, 2026. The copyright holder for this preprint (which was not certified by peer review) is the author/funder, who has granted bioRxiv a license to display the preprint in perpetuity. It is made available under a CC-BY 4.0 International license.

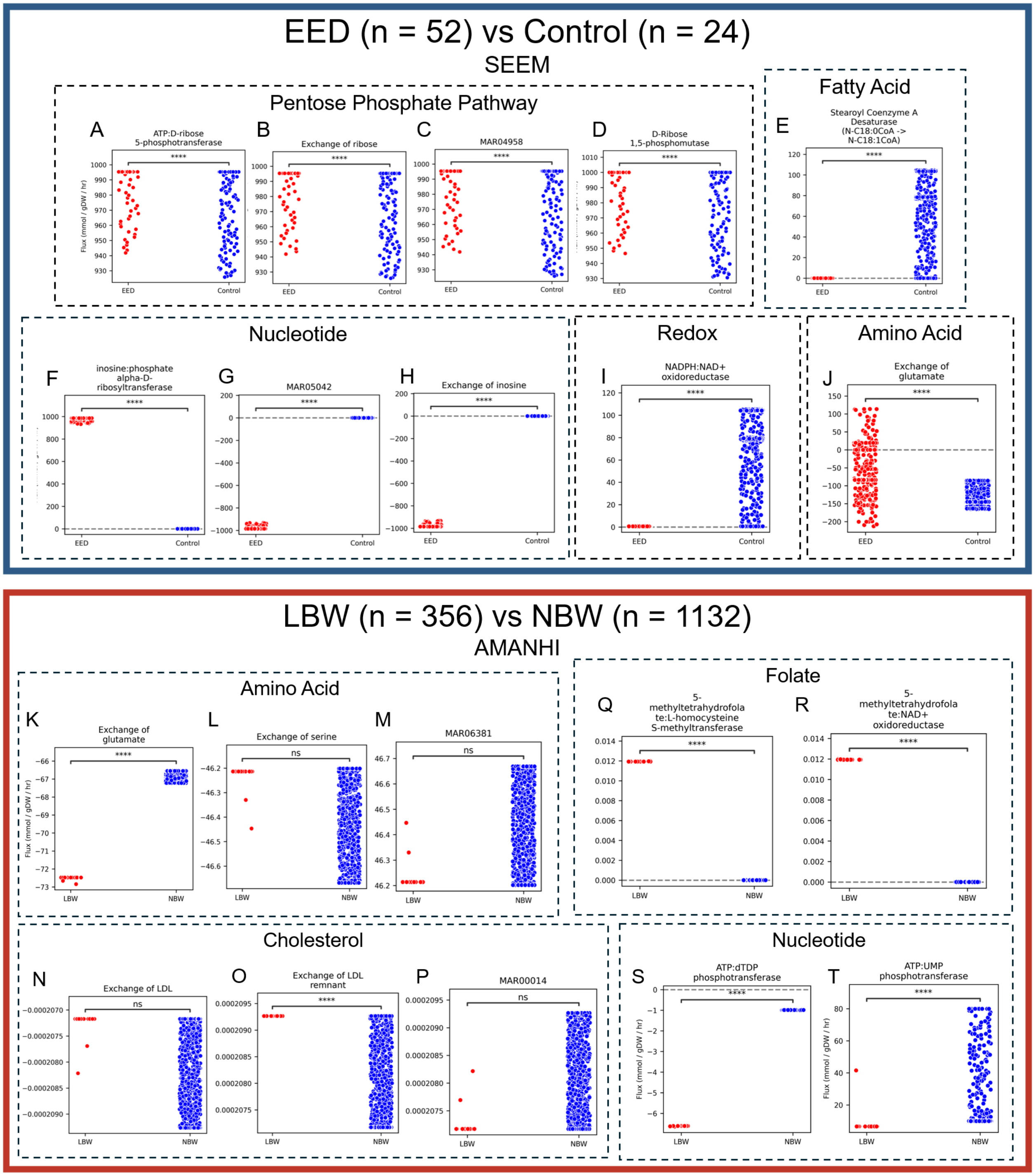

bioRxiv preprint doi: https://doi.org/10.64898/2026.10.25.760166; this version posted October 30, 2026. The copyright holder for this preprint (which was not certified by peer review) is the author/funder, who has granted bioRxiv a license to display the preprint in perpetuity. It is made available under a CC-BY 4.0 International license.

Supplementary Figure 2. Altered Amino Acid and Nucleotide Metabolism Differentiates EED from Controls and LBW from NBW.
Jitter plots showing the top 10 reactions identified by our Reaction Inclusion by Parsimony and Transcript Distribution (RIPTiDe) and Linear Discriminant Analysis framework. Our framework was applied to SEEM duodenal transcriptomics (A-J) and to AMANHI maternal plasma proteomics from visit 2 (24-28 weeks) (K-T). The top 10 reactions were categorized into subsystems by a gastroenterologist based on their expert opinion as to each reaction's biochemical function. In each plot, the x-axis describes the altered reaction between 1) EED (n = 52) or 2) LBW (n = 356) and 1) controls (n = 24) or 2) NBW (n = 1132). The y-axis shows the flux values sampled from the context-specific metabolic network reconstructions produced by RIPTiDe. A two-sided Mann-Whitney U-test was used to assess the significance of differences in flux distributions. Note that the scale of the y-axes varies between plots and is shown primarily for illustrative purposes.

Both sets of discriminating reactions implicate Nucleotide Metabolism (F, G, H, S, T) and Amino Acid Metabolism (J, K, L, M).

n.s. = not significant ($p >= 0.05$)
* = $0.01 < p < 0.05$
** = $0.001 < p < 0.01$
*** = $0.0001 < p < 0.001$
**** = $0.00001 < p < 0.0001$

Abbreviations:
EED = Environmental Enteric Dysfunction; SEEM = Study of Environmental Enteropathy and Malnutrition; LBW = low birth weight; NBW = normal birth weight; AMANHI = Alliance for Maternal and Newborn Health Improvement; MAR04958 = ribose [c] ⇔ ribose [e]; MAR05042 = inosine [c] ⇔ inosine [e]; MAR06381 = H^+ [c] + Na^+ [e] + serine [e] ⇒ H^+ [e] + Na^+ [c] + serine [c]; MAR00014 = LDL [e] ⇒ LDL remnant [e] + 680 cholesterol [e] + 1515 cholesterol-ester pool [e]; ATP = adenosine triphosphate; NADPH = nicotinamide adenine dinucleotide phosphate; NAD+ = nicotinamide adenine dinucleotide; LDL = low density lipoprotein; H^+ = hydrogen ion; Na^+ = sodium ion

Definitions: LBW = birthweight < 2.5 kg; NBW = birthweight ≥ 2.5 kg

Variable	FDR q-value				
	**Chronic Hypertension (n = 140 yes, n = 2,259 no)**	**Diabetes (n = 19 yes, n = 2,383 no)**	**Epilepsy (n = 2 yes, n = 2,400 no)**	**Tuberculosis (n = 32 yes, n = 2,370 no)**	**Thyroid Disease (n = 12 yes, n = 2,390 no)**
**Birth Outcomes and Infant Anthropometrics at Birth**					
Pregnancy Outcome	0.35	0.35	1.00	0.96	0.13
Term of Delivery	0.35	0.09	1.00	0.96	0.34
Birthweight	0.69	0.68	1.00	0.57	0.13
Birth Phenotype	0.35	0.35	1.00	1.00	0.47
**Infant Anthropometrics at 24-Month Follow-Up**					
Stunting	0.35	0.09	1.00	0.96	0.34
Wasting	0.35	1.00	1.00	0.57	0.62

bioRxiv preprint doi: https://doi.org/10.64898/2026.10.25.760166; this version posted October 30, 2026. The copyright holder for this preprint (which was not certified by peer review) is the author/funder, who has granted bioRxiv a license to display the preprint in perpetuity. It is made available under a CC-BY 4.0 International license.

Supplementary Table 1. Maternal Comorbidities are Not Significantly Associated with Adverse Birth Outcomes or Infant Growth Outcomes at 2 Years.
The association between maternal comorbidities (history of chronic hypertension (n = 140), diabetes (n = 19), epilepsy (n = 2), tuberculosis (n = 32), or thyroid disease (n = 12)) with adverse birth outcomes and infant anthropometrics at 2 years of age is presented. Each variable presented is categorical; numerical variables such as birth weight were discretized into clinically meaningful categories prior to statistical analysis. P-values were calculated by a Fisher's Exact Test with probabilities approximated via Monte Carlo simulation, then adjusted by the Benjamini-Hochberg procedure. After adjustment, no significant ($q < 0.05$) associations were found.

Abbreviations: FDR = false discovery rate

bioRxiv preprint doi: https://doi.org/10.64898/2026.10.25.760166; this version posted October 30, 2026. The copyright holder for this preprint (which was not certified by peer review) is the author/funder, who has granted bioRxiv a license to display the preprint in perpetuity. It is made available under a CC-BY 4.0 International license.

Proxy Outcome	OOB Balanced Accuracy (%)
SGA (n = 674) vs AGA (n = 1305)	54.3%
Preterm (n = 368) vs Term (n = 2021)	51.5%
LBW (n = 489) vs NBW (n = 1491)	52.0%
Stunted (n = 928) vs Not Stunted (n = 857) at 2 Years	56.9%
Wasted (n = 209) vs Not Wasted (n = 1569) at 2 Years	50.0%

bioRxiv preprint doi: https://doi.org/10.64898/2026.10.25.760166; this version posted October 30, 2026. The copyright holder for this preprint (which was not certified by peer review) is the author/funder, who has granted bioRxiv a license to display the preprint in perpetuity. It is made available under a CC-BY 4.0 International license.

Supplementary Table 3: Maternal Metadata is an Insufficient Predictor of Child Growth Outcomes.

Five random forest models were trained to predict each of the five proxy outcomes using only maternal metadata (not using proteomics data). The OOB balanced accuracies are presented in the table as an estimate of the models' accuracies for predicting the outcomes for unseen participants, adjusting for class imbalance. The balanced accuracy score has a minimum value of 0% and a maximum value of 100%, with 50% being the expected accuracy of a blind estimator. The OOB balanced accuracies attained by our random forest models are consistently low – indicative that maternal metadata is an insufficient predictor of any of the proxy outcomes.

Abbreviations: OOB = out-of-bag; SGA = small for gestational age; AGA = appropriate for gestational age; LBW = low birth weight; NBW = normal birth weight

Definitions: SGA = birthweight < 10th percentile for gestational age; AGA = birthweight ≥ 10th percentile for gestational age; LBW = birthweight < 2.5 kg; NBW = birthweight ≥ 2.5 kg; Stunted at 2 years = length-for-age Z-score < -2.0 at 2 years of age; Not stunted at 2 years = length-for-age Z-score ≥ -2.0 at 2 years of age
